# Single-cell sequencing reveals the expansion and diversity of T cell subsets in the bone marrow microenvironment of chronic myeloid leukemia

**DOI:** 10.1016/j.gendis.2025.101626

**Published:** 2025-04-04

**Authors:** Chenjian Zhuo, Xin Dong, Xueya Zhao, Weiru Wu, Hao Zhou, Jing Feng, Lingbo Liu, Mingqian Feng, Chunjiang He, Yu Hou

**Affiliations:** a College of Biomedicine and Health, Hubei Hongshan Laboratory, College of Informatics, College of Life Science and Technology, Huazhong Agricultural University, Wuhan, Hubei 430070, China; b School of Basic Medical Sciences, Wuhan University, Wuhan, Hubei 430071, China; c School of Basic Medical Sciences, Chongqing Medical University, Chongqing 400016, China; d Department of Clinical Hematology, Third Military Medical University (Army Medical University), Chongqing 400016, China; e Department of Hematology, Union Hospital, Tongji Medical College, Huazhong University of Science and Technology, Wuhan, Hubei 430070, China; f School of Computer Science, Wuhan University, Wuhan, Hubei 430072, China; g College of Biomedicine and Health, College of Life Science and Technology, Huazhong Agricultural University, Wuhan, Hubei 430070, China

**Keywords:** Chronic myeloid leukemia, T cell receptor, Single-cell sequencing, T cell, Cell-cell communication

## Abstract

The immune microenvironment plays an important role in leukemia treatment. However, a specific single-cell profiling of the immune alteration in bone marrow of chronic myeloid leukemia (CML) patients is still lacking. We performed multi-level single-cell sequencing to systematically decipher the bone marrow T cell atlas of CML patients. The results exhibited extensive changes of T cells, including the decreased CD4 T cells and increased CD8 T cells in the CML bone marrow. Subpopulation analysis revealed a significant increase of CD8 terminal effector (TE) cells and a significant decrease of CD4 naïve T cells. T cell receptor sequencing showed that the overall diversity of the T cell receptor repertoire was reduced in CML, with the exception of the CD8 TE cell. In addition, CD8 TE cells were the main source of gene expression differences in CD8 T cells. Intercellular communication analysis revealed the altered interaction between CD8 TE and other non-T cells in CML, including neutrophil subtype, indicating the potential regulation of bone marrow microenvironment cells on CD8 TE dynamics. Collectively, our work characterises the alteration of T cell subsets in CML patients at multiple single-cell levels, providing a valuable resource for understanding the immune microenvironment and developing new immune strategies for CML therapy.

## Introduction

Chronic myeloid leukemia (CML) is a hematopoietic malignancy driven by the fusion gene *BCR-ABL1*. Gene fusions mostly occur in hematopoietic stem cells. Though tyrosine kinase inhibitors can be used to arrest disease progression and achieve deep molecular remission,^1^ relapse and drug resistance remain obstacles to CML treatment.^2^ The immune system has been reported to affect patient treatment and prognosis.^3^^,^^4^ Deep molecular response is associated with increased CD8^+^ T cells.^5^ High CD62L^+^ T cells and low sCD62L^+^ were associated with patient response to nilotinib.^6^ Therefore, exploring the changes in the immune microenvironment of CML patients is of great significance for evaluating therapy.

A low-cell-throughput approach based on flow cytometry has been used to report immune alterations in the bone marrow of CML, revealing a decrease in anti-cancer immune cells and an increase in immunosuppressive cells.^7^ At the single-cell level, several studies have revealed the composition of bone marrow mononuclear cells or peripheral blood mononuclear cells of CML patients, but have not elucidated the status of T cells or T cell receptors, nor considered the interaction between the non-immune cells and T cells.^8^, ^9^, ^10^ Although immune cell changes in bone marrow malignancies have been extensively analysed in acute myeloid leukemia,^11^, ^12^, ^13^ a systematic analysis focusing on the CML immune microenvironment in single-cell resolution is still lacking.

Here, we combined single T cell, single-cell T cell receptor (TCR), and single-cell whole bone marrow sequencing to analyse the bone marrow immune microenvironment of newly diagnosed CML patients and healthy controls. We identified the expansion and diversity of T cell subsets in CML and demonstrated the potential communication between non-immune cells and T cell subsets in the bone marrow microenvironment. Our work is the first to systematically define the CML-specific T cell population in single-cell resolution, provideing new insights into potential immunotherapy strategies for CML.

## Methods

### Sample collection

Bone marrow samples of five newly diagnosed CML chronic phase patients and three healthy donors were obtained from the Department of Hematology of Southwest Hospital (Chongqing, China). Informed consent was acquired according to protocols approved by the Ethics Committee of the Army Medical University. All patients gave informed consent according to the Declaration of Helsinki. Bone marrow cells were isolated by centrifugation at 300 *g* and 4 °C for 5 min after mixing 3 volume red blood cell lysis buffer (Solarbio life science R1010) and samples containing 1 volume IMDM medium (Gibco, 12440053) on ice for 10 min. Cells were stained in FACS sorting buffer containing Dulbecco’s phosphate-buffered saline (Solarbio life science, D1040) with 2% fetal bovine serum, 1% penicillin–streptomycin (Gibco, 15140122), and 1 mmol L^−1^ EDTA (Beyotime Biotechnology, C0196) with FITC anti-human CD3 (clone HIT3a; BioLegend, 300306) at 4 °C for 30 min. After staining, cells were washed once and resuspended in FACS sorting buffer and sorted on BD FACS Aria II.

### Single-cell library preparation and sequencing

The viability and concentration of cell suspension were detected using an automated cell counter (Bio-Rad, 145–0102) after sorting and adding filtered trypan blue. To maximize the achievement of desired cell recovery, the cell stock concentration should be 700–1200 cells/μL, and viability should be between 85% and 95% to improve the recovery rate. The cells were resuspended in cold Dulbecco’s phosphate-buffered saline containing 0.04% bovine serum albumin based on the results of the cell counter and the recommended cell concentration. Total bone marrow mononuclear cells (viability was between 85% and 95%) from healthy donors or CML patients were loaded onto the 10x Genomics chromium single-cell platform, and libraries were constructed using a Chromium single cell 3′ library kit V3 (10x Genomics, 1000075). Libraries of CD3^+^ cells from healthy donors or patients with CML were constructed using the Chromium single cell 5′ library (10x Genomics, 1000165) and V(D)J enrichment (10 × Genomics, 1000005) kits. The quality of the complementary DNA, cDNA post target enrichment, and final libraries were assessed using Agilent Bioanalyzer high-sensitivity chips on an Agilent Bioanalyzer 2100. Sequencing was performed with Illumina (NovaSeq 6000) according to the manufacturer’s instructions (Illumina).

### Single-cell RNA sequencing data processing

Cellranger (v3.1.0) was used to map reads to reference genome (GRCh38.p12) and generate a gene expression matrix. After obtaining the expression matrix, subsequent analysis was performed using Seurat.^14^ The specific steps were as follows. First, we retained cells with more than 200 genes and less than 4000 genes, requiring that the proportion of mitochondrial genes should not exceed 15%. Then, we use the NormalizeData function to normalize the original count matrix and the FindVariableFeatures function to find the top 2000 highly variable genes. The ScaleData function was used to perform z-score conversion on the data so that the RunPCA function could reduce the dimensionality of the data. Since the cells come from different samples, batch effects need to be removed. Here, we used the harmony package^15^ to remove the batch effect and then used the RunUMAP and FindNeighbors functions to perform dimensionality reduction visualization and unsupervised clustering of cells, respectively. FindAllMarkers was used to find differential genes between cell clusters. Finally, based on the currently known cell markers, we annotated the cell types of all clusters.

### Differential analysis and pathway enrichment

To compare the expression difference of cells between CML and healthy controls, we utilized the pseudo-bulk method for differential analysis. Specifically, we first extracted the expression matrix corresponding to the cell type and summed the cell counts of each sample to obtain the pseudo bulk expression matrix of each sample. Then DESeq2^16^ was used for differential analysis, and genes with *p* value < 0.05 and |log_2_FC| ≥ 0.25 were regarded as differential genes.^17^ Differential genes were enriched for pathways using the clusterProfiler.^18^ The DotPlot and VlnPlot functions from Seurat were used to draw the gene expression dot and violin chart, respectively. The ggplot2 package^19^ was used to draw the pathway bubble chart.

### Calculation of cytotoxicity scores

Cytotoxic genes (NKG7, CCL4, CST7, PRF1, GZMA, GZMB, IFNG, CCL3, and FGFBP2) were used to calculate the cytotoxic scores using the AddModuleScore function.^20^, ^21^, ^22^ A significance level of the differences in the cytotoxic scores in CD4 T/CD8 T cells between CML and healthy controls was evaluated using a *t*-test.

### Flow cytometry

The percentages of CD4 naïve T cells and CD8 terminal effector (TE) cells in additional five CML chronic phase patients and three healthy bone marrow samples were detected using a flow cytometer (Beckman DxFLEX B5-R3-V5). Antibodies included PC7 anti-human CD3 (clone UCHT1, Beckman, 6607100), APC anti-human CD8 (clone B9.11, Beckman, IM2469), ECD anti-human CD45RO (clone UCHL1, Beckman, B49192), PE anti-human CD197 (clone G043H7, Beckman, B30632), FITC anti-human CD95 (cloneDX2,BD, 340479), Pacific Blue anti-human CD4 (clone 13B8.2, Beckman, B49197), Krome Orange anti-human CD45 (clone J33, Beckman, B36294), APC-A750 anti-human CD28 (clone CD28.2, Beckman, B08757). CD4 naïve T cells were identified using CD3^+^CD4^+^CD45RO^−^CCR7^+^, and CD8 TE cells were identified using CD3^+^CD8^+^CD45RO^+^CCR7^−^CD95^−^CD28.

### Deciphering cell–cell communication

CellphoneDB^23^ was used to predict the communication of signals between cells. First, we used the statistical analysis module in CellphoneDB to predict the cell-to-cell communication in each sample with default parameters. To identify the differential interaction frequency between CML and healthy controls, we normalized the frequency of cell-to-cell communication in all samples, that is, dividing the frequency of each cell communication by the sum of the frequency of cell communication in this sample. Then, all data were combined into a matrix, analyzed for differences using *t*-tests, and visualized using the pheatmap package.^24^ Similarly, to obtain the ligand-receptor pairs that differed between CML and healthy controls, we combined the mean expression levels of ligand-receptor pairs in each sample into a matrix and used the *t*-test for differential analysis.

### Single-cell TCR sequencing analysis

TCR full-length sequencing was generated by 10x Genomics Chromium Single-Cell V(D)J Enrichment kit. The Cell Ranger VDJ pipeline assembled TCR sequences from enriched libraries. Then, the R packages immunarch^25^ and scRepertoire^26^ were used to analyze TCR. Specifically, the repExplore function was used to count and compare sample clonal shapes, the repOverlap function was used to overlap TCR clones between samples, repClonality was used to evaluate the proportion of the most abundant TCR clone in the sample, and the repDiversity function was used to calculate the TCR clone diversity Hill index. In addition, scRepertoire’s clonalDiversity function was used to calculate the Shannon entropy index. Finally, the combineExpression function of scRepertoire was used to combine gene expression and TCR clone types.

### Correlation analysis

To measure the association of T cell subsets with non-T cells in the bone marrow microenvironment, the proportion of T cell subsets and non-T cells in each sample was extracted, and the Spearman correlation coefficient was calculated.

### Cell trajectory analysis

Monocle2^27^ was used to investigate the potential differentiation trajectory of neutrophils. First, the differentialGeneTest function was used to generate differential genes. Then, dimension reduction and cell ordering were carried out based on the DDRTree method. Finally, the cell trajectory was visualized.

### Transcription factor regulon analysis

The analysis of the regulatory network and regulon activity was performed by pySCENIC.^28^ The regulon activity (measured in AUC) was analyzed by the AUCell module of the pySCENIC, and the active regulons were determined by AUCell default threshold.

### Statistical analysis

All statistical analyses were performed using GraphPad Prism (GraphPad Software, La Jolla, CA) and R package (https://www.r-project.org/).

## Results

### Profiling of bone marrow T cell composition in CML patients

**Figure 1:**
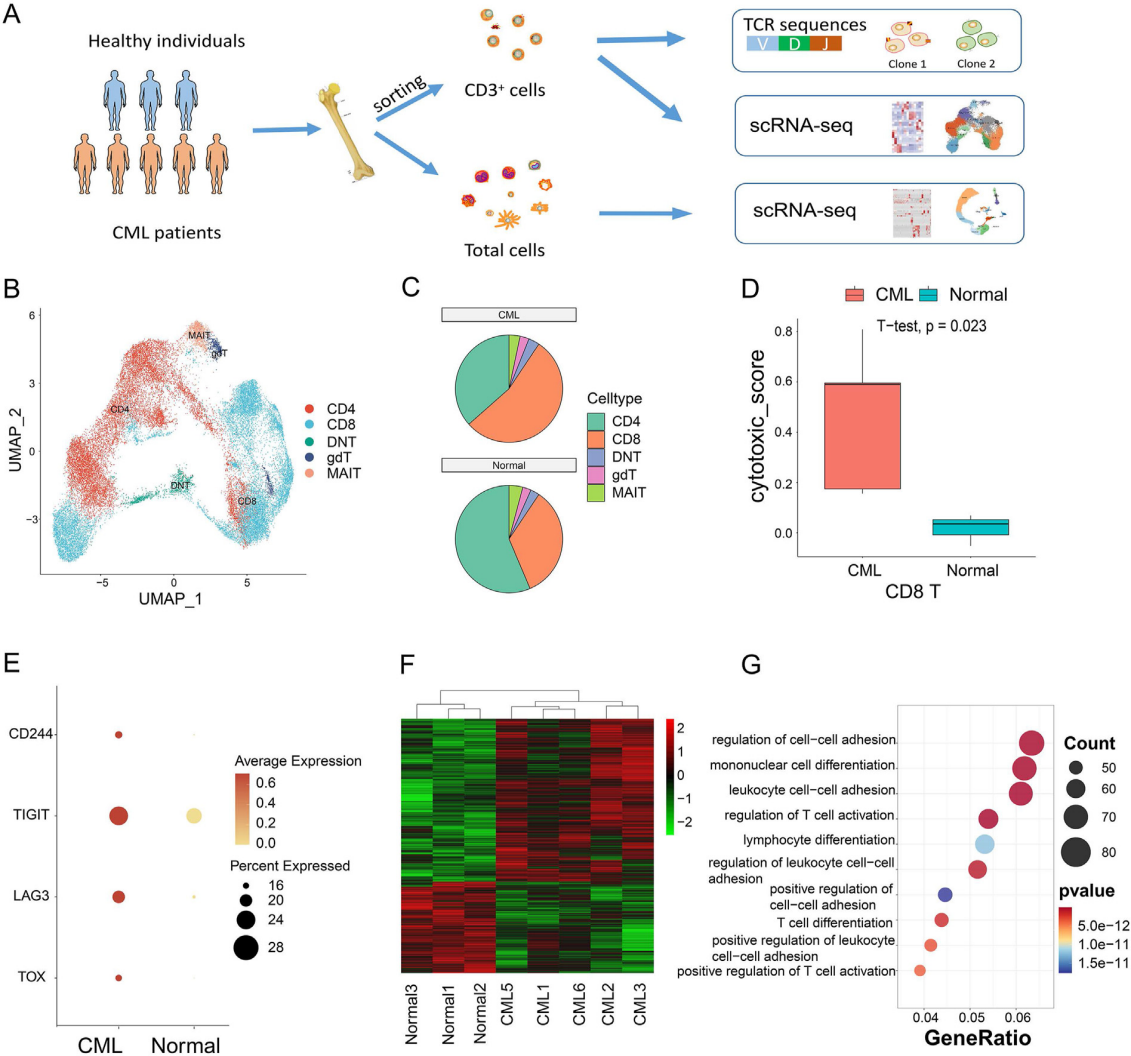
Composition and functional variation of T cells in CML. **(A)** Overview of this study. **(B)** The UMAP diagram showing the distribution of CD4, CD8, DNT, gdT, and MAIT in the samples. **(C)** The pie chart showing the ratio of T cells in CML and healthy samples. **(D)** The boxplot showing the cytotoxicity scores of CD8 T cells between CML and healthy samples. **(E)** The dot plot showing the expression of immunosuppression genes in CD8 T cells between CML and healthy samples. The size of the circle indicates the ratio of cells expressing these genes. **(F)** The heat map showing the differentially expressed genes of CD8 T cells between CML and healthy controls. **(G)** The bubble plot showing the biological functions of differentially expressed genes in CD8 T cells. CML, chronic myeloid leukemia; DNT, double negative T cells; gdT, γ-δ T cells; MAIT, mucosal-associated invariant T cells.

To understand the unique immune response of CML, we collected bone marrow samples from five CML patients and three healthy control samples and sorted CD3^+^ cells (Fig. 1A and Table S1). After quality control, 35,230 T cells were detected, of which 20,461 cells were from CML patients and 14,769 cells were from healthy controls. After removing batch effects and dimensionality reduction, 35,230 cells were clustered into 19 clusters (Fig. S1A and B). Using known cell markers (Table S2), we identified two classes of conventional T cells and three classes of unconventional T cells. Two classes of conventional T cells were CD4T and CD8T, accounting for 36.51% and 54.1% of bone marrow T cells in CML patients and 56.4% and 34.2% of bone marrow T cells in healthy controls, respectively. Three classes of unconventional T cells include double negative T cells, γ-δ T cells, and mucosal-associated invariant T cells, accounting for 3.32%, 2.75%, and 3.32% of T cells respectively in CML patients and accounting for 2.7%, 2.55%, and 4.14% of T cells respectively in the control group (Fig. 1B, C; Fig. S1C). Considering the number of cells, CD8 T cells showed an expansion trend in CML, while CD4 T cells were the opposite.

Next, we used AddModuleScore to calculate cytotoxicity marker (NKG7, CCL4, CST7, PRF1, GZMA, GZMB, IFNG, CCL3, and FGFBP2) scores to evaluate the cytotoxicity of CD4 T cells and CD8 T cells. The results showed that the cytotoxicity of CML CD8 T cells was significantly stronger than that of the healthy sample (*p* = 0.023) (Fig. 1D). As T cell toxicity may be linked to cell exhaustion,^29^ we checked the expression of T cell immunosuppressive markers in CD8 T cells and found that the expression of four immunosuppressive markers TOX, LAG3, TIGIT, and CD244 were higher in CML patients (Fig. 1E), indicating that CD8 T cells may gradually lose their effector function due to long-term exposure to antigens and transform into exhausted T cells, thereby inhibiting the immune effect.^30^ Although CML CD4 T cells were also slightly more cytotoxic than CD4 T cells from healthy samples, the difference was not significant *(p* = 0.082) (Fig. S2A). Previous reports have shown that T cell toxicity is related to recognizing and killing infected or malignant cells and mediating antiviral and anti-tumor immunity,^31^ indicating that the same trend exists for T cells in CML patients.

We further explored differences in gene expression of CD4 T cells and CD8 T cells between CML and control samples. In CD8 T cells, we found 990 genes up-regulated and 556 genes down-regulated in CML (Fig. 1F and Table S3). These differential genes mainly regulate biological functions such as leukocyte cell adhesion, monocyte differentiation, and regulation of T cell activation regulation (Fig. 1G). In CD4 T cells, we observed 791 genes up-regulated and 335 genes down-regulated in CML (Fig. S2B and Table S4). Functional enrichment showed that these differential genes were also enriched in intercellular adhesion of leukocytes, leukocyte migration, monocyte migration, *etc* (Fig. S2C). These results indicate that the gene expression of CD4T and CD8T cells is altered between CML and control samples and may be involved in changes in cell function.

### CD8 TE and CD4 naïve T cells exhibit significant alterations in CML bone marrow

**Figure 2:**
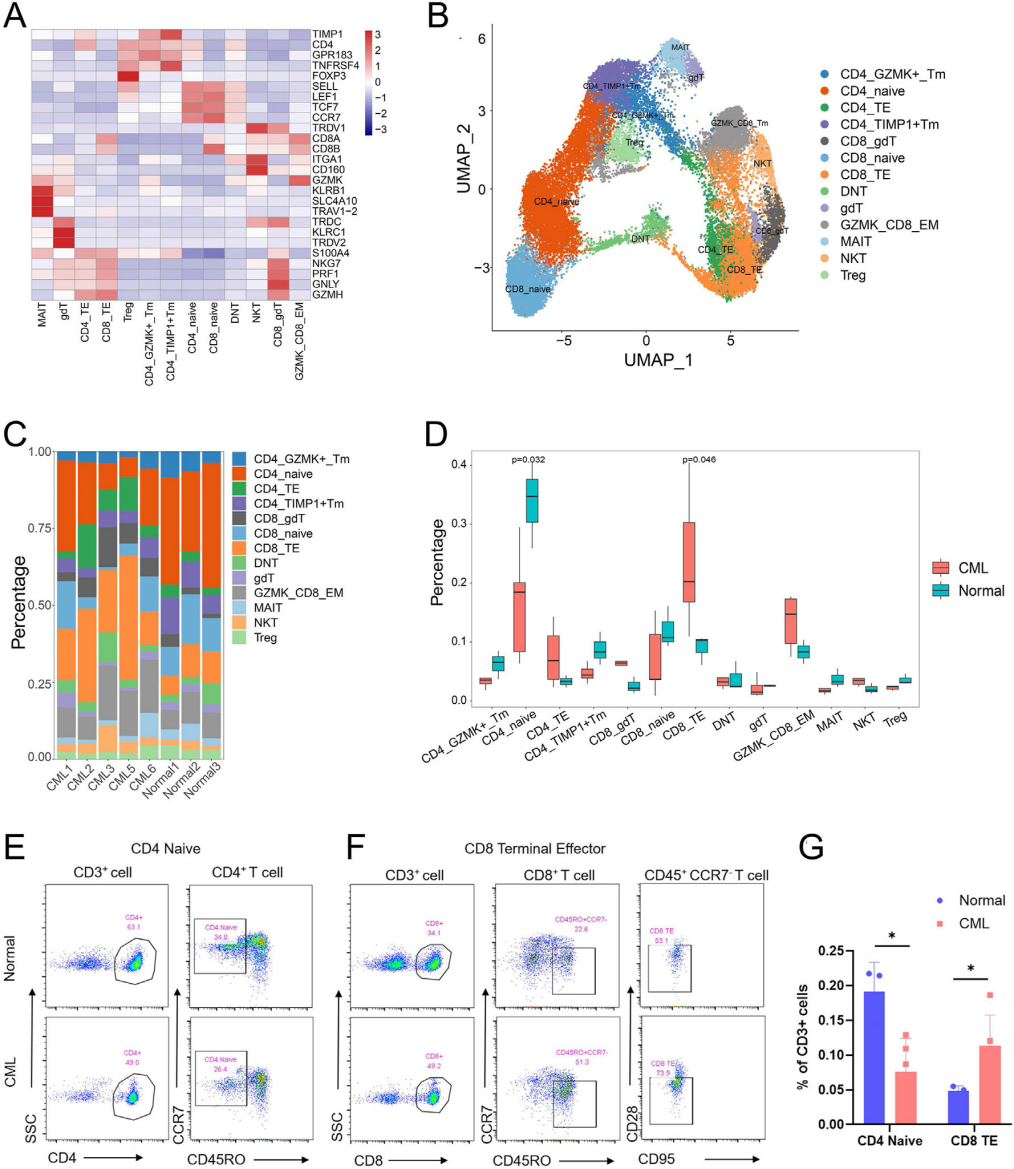
Differential T cell subpopulations in CML and healthy controls. **(A)** The heatmap showing the expression of classic T cell subset markers. **(B)** The UMAP diagram showing the distribution of T cell subsets. **(C)** The histogram showing the proportion of T cell subsets in each sample. **(D)** The boxplot showing the difference in the composition of each subtype between CML and healthy controls. *t*-test was used to define the significance. **(E)** Flow cytometry analysis of CD4 naïve T cells in healthy samples (*n* = 3) and CML patients (*n* = 5). **(F)** Flow cytometry analysis of CD8 TE cells in healthy samples (*n* = 3) and CML patients (*n* = 5). **(G)** Frequency of CD4 naïve T cell and CD8 TE cell subsets in healthy samples and CML patients. Unpaired *t*-test with mean ± standard deviation. ∗*p* < 0.05. CML, chronic myeloid leukemia; TE, terminal effector.

Using known cell markers (Table S2), T cell subsets were further annotated. CD4 naïve and CD8 naïve highly express classic markers such as SELL, LEF1, and CCR7.^20^^,^^32^ Memory T cells express low levels of naïve T cell markers and are divided into CD4 TIMP1^+^ Tm and CD4 GZMK^+^ Tm based on their specific expression of TIMP1 and GZMK. GZMK CD8 EM and NKT cells highly express CD160.^33^ CD8 TE and CD4 TE cells highly express cytotoxic markers such as NKG7, GNLY, GZMH, and PRF1. In addition, among CD8 T cells, we found a group of cells expressing γ-δ T cell markers TRDC, TRDV1, *etc*.,^34^ which was annotated as CD8 γ-δ T cells. In total, we detected 13 T cell subsets in CML and healthy bone marrow (Fig. 2A, B). After identifying T cell subpopulations, we studied the proportional distribution of these subpopulations. The results showed that the distribution of CD4 naïve, CD8 TE, and CD4 TE cells differed between CML and healthy bone marrow (Fig. 2C). We further tested its distribution using a *t*-test. The results showed that no significant difference in CD4 TE was observed between CML and control samples. In contrast, the proportion of CD8 TE cells was significantly increased in CML (*p* = 0.046), and the proportion of CD4 naïve cells was significantly decreased in CML (*p* = 0.032), which may indicate that CD8 TE and CD4 naïve cells are mainly involved in the CML immune microenvironment changes (Fig. 2D). To further validate our results, we performed a flow cytometry analysis of CD4 naïve T cells and CD8 TE cells in the bone marrow of CML and healthy samples. We found that the proportion of CD4 naïve T cells in CML patients was significantly lower than in the healthy samples, while CD8 TE cells were significantly increased, which is consistent with single-cell sequencing analysis (Fig. 2E–G).

### CML impairs T-cell diversity

**Figure 3:**
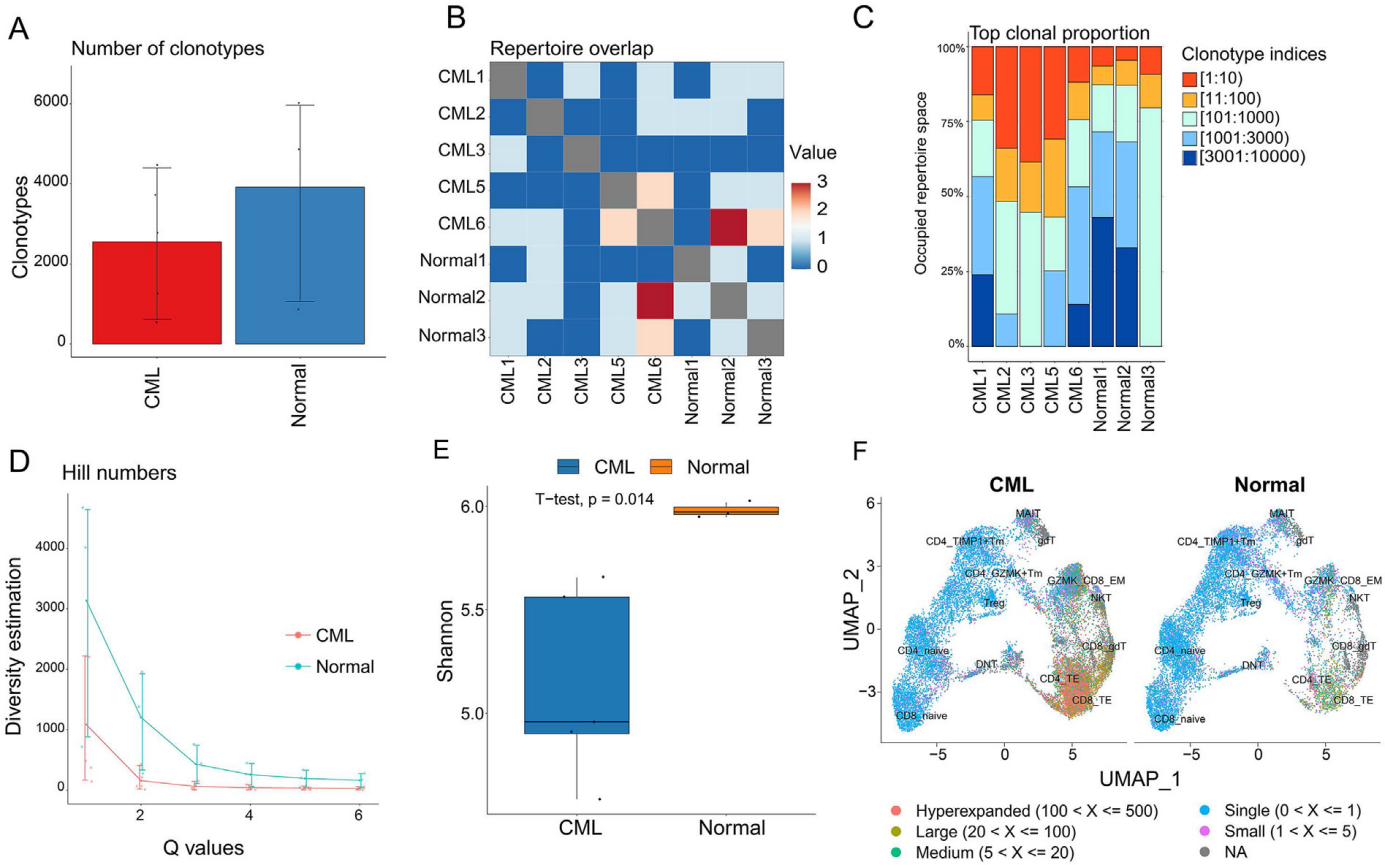
CML shows reduced TCR diversity. **(A)** The histogram showing the number of TCR clones detected in CML and healthy controls. **(B)** The heatmap showing the overlap of clonotypes between samples. **(C)** The histogram showing the expansion of clonotypes in all samples. CML exhibited stronger clonal expansion. **(D, E)** The Hill and Shannon entropy index showing differences in TCR diversity between CML and healthy controls. **(F)** The UMAP plot showing major enriched cell populations for TCR clonal expansion. TCR, T cell receptor; CML, chronic myeloid leukemia.

To explore the potential T cell regulation in CML, we performed single-cell TCR sequencing on CD3^+^ cells from CML and healthy bone marrow samples (Fig. 1A). The results showed that an average of 2555 TCR clonotypes were detected in CML, whereas an average of 3915 clonotypes were detected in control samples (Fig. 3A). Only a very small number of TCR clones were identical across all eight samples, suggesting significant TCR specificity between donors (Fig. 3B). We examined the distribution of the most abundant clones in each sample and found that the most abundant clonotype was significantly increased in CML patients (Fig. 3C). Next, to provide a more comprehensive quantification of TCR diversity, we calculated two indexes (Hill number and Shannon entropy) for each sample. The results showed that the diversity of TCRs in CML was significantly less than that in control samples (Fig. 3D, E). Since previous reports revealed that changes in cell proportions in bone marrow may affect the composition of TCR complexes in T cells,^35^ we further mapped TCR clones onto the T cell subpopulations and identified cell types with clonal expansion. The results showed that clonal expansion mainly occurred in CD4 TE and CD8 TE cells (Fig. 3F). Overall, these results demonstrate that TCR diversity is reduced in CML.

### CD8 TE cells demonstrate CML-specific gene expression alterations

**Figure 4:**
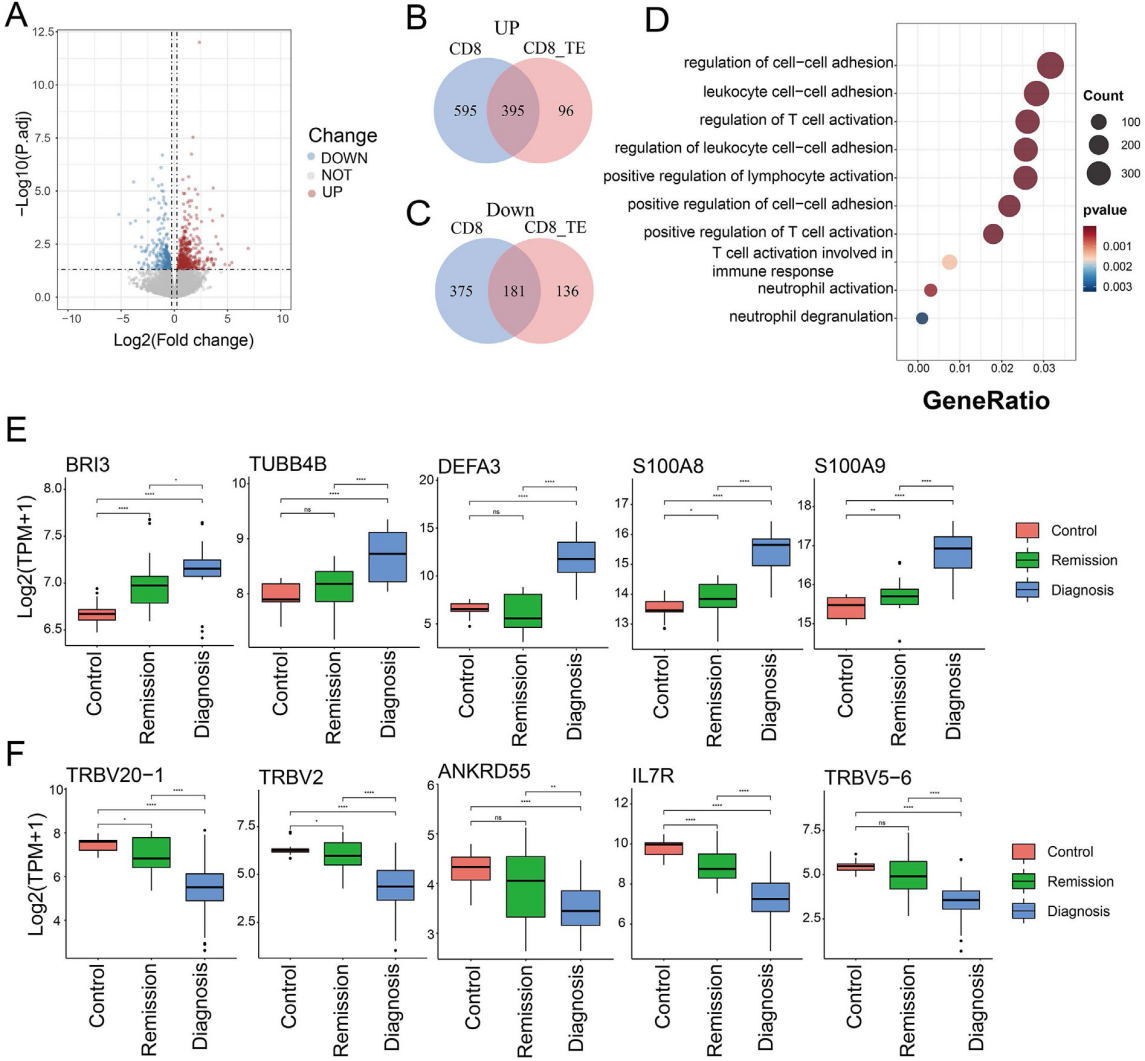
CD8 TE cell demonstrates CML-specific gene expression. **(A)** The volcano plot showing differential genes of CD8 TE cells between CML and healthy controls. **(B)** The Venn diagram showing the overlap of up-regulated genes in CD8 TE and CD8 T cells in CML. **(C)** The Venn diagram showing the overlap of down-regulated genes in CD8 TE and CD8 T cells in CML. **(D)** The bubble plot showing the biological function enrichment of differential genes in CD8 TE cells. **(E, F)** The boxplots showing the differential gene expression between clinical newly diagnosed CML patients, CML patients in remission, and healthy control samples based on bulk RNA sequencing data. CML, chronic myeloid leukemia; TE, terminal effector.

Both T cell sequencing and TCR analysis results showed that CD8TE cells may undergo significant changes in CML. We further analyzed the differential genes of CD8TE between CML and control samples, and the results showed that 491 genes were up-regulated and 317 genes were down-regulated in CD8 TE of CML (Fig. 4A and Table S5). In addition, these differential genes had considerable overlap with those of CD8 T cells between CML and control samples, including 39.9% up-regulated genes and 32.5% down-regulated genes, which was the highest among all CD8 T cell subtypes. Another 232 differentially expressed genes were specifically present in CD8 TE cells (Table S6). This indicates that a large part of the gene expression changes in CD8 T cells in CML originate from CD8 TE cells (Fig. 4B, C). Gene enrichment analysis showed that differentially expressed genes in CD8 TE cells were involved in important biological functions, such as regulating T cell activation and leukocyte cell–cell adhesion (Fig. 4D).

To explore the genes in CD8 TE cells with an important impact on CML, we obtained bulk RNA sequencing data, including healthy donors, CML patients, and patients in remission from the GEO database (GSE144119), and detected the expression level of those differential genes identified in CD8 TE cells. We found that several genes showed the same trend in bulk data (Fig. 4E, F). For example, IL7R, which is downregulated in CML CD8 TE cells, also has reduced expression in CML bulk samples, which is consistent with previous studies revealing that IL7R signaling can prevent leukemogenesis.^36^ High expression levels of DEFA3 were relevant to a poor prognosis in children with acute myeloid leukemia.^37^ Moreover, the biologic effects of S100A8/9 via both TLR4 and RAGE on hematopoietic stem cells contribute to the selection and expansion of leukemic clones by excretion of proinflammatory cytokines and/or immune regulation.^38^ In summary, by analyzing paired single-cell TCR and T cell single-cell RNA profiles, we found that the expansion of CD8 TE cells in CML and a large number of differential genes in CD8 TE were enriched in important signaling pathways, indicating that CD8 TE cells may play an important role in CML.

### Bone marrow microenvironment cells may influence the expansion of CD8 TE cells in CML

To explore the potential regulation of other non-T cells in CML bone marrow on the expansion of CD8 TE cells, we performed single-cell sequencing for whole bone marrow cells (Fig. 1A and Table S1). After removing samples with poor sequencing quality, a total of 33,959 whole bone marrow cells were detected, including 23,037 cells from five CML patients and 10,922 cells from two healthy controls. Based on marker gene expression (Table S2), we classified these cells into 16 cell types (Fig. S3A and B).

**Figure 5:**
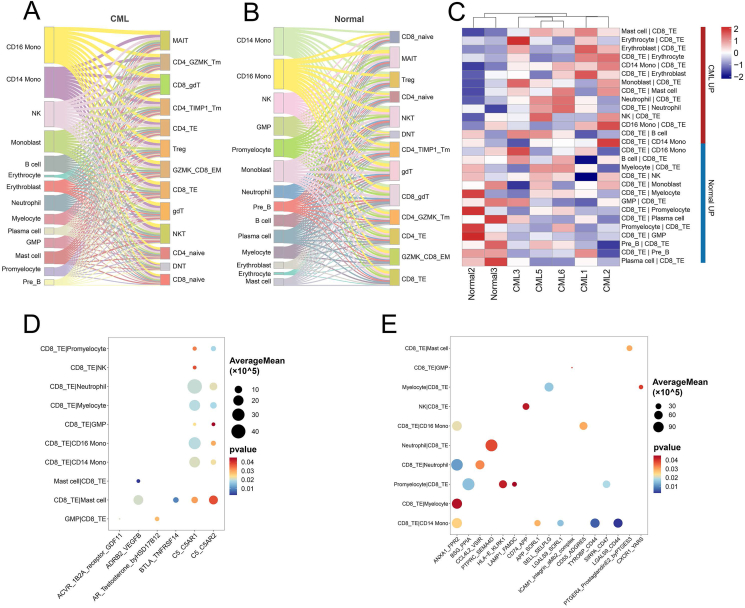
Bone marrow non-T cells are communicated with CD8 TE cells. **(A, B)** The Sankey plot showing numerous interactions between microenvironment cells and T cell subsets in CML and healthy controls. **(C)** The heatmap showing the interaction frequency between CD8 TE and bone marrow cells in CML and healthy donors. **(D, E)** The bubble plots showing the up-regulated (D) and down-regulated (E) ligand-receptor pairs between CD8 TE and bone marrow cells in CML patients. CML, chronic myeloid leukemia; TE, terminal effector.

Then, we integrated the T cell subsets and whole bone marrow cells to calculate the cell–cell interactions using cellphoneDB.^23^ We observed numerous ligand-receptor interactions between non-T cells and T cell subsets in both CML and healthy controls (Fig. 5A, B). Analysis of interaction frequency showed significant differences between CML and control samples. For example, the interaction frequency between CD8 TE and pre B cells was reduced in CML and the interaction frequency between CD8 TE and neutrophil was elevated in CML (Fig. 5C). We further analyzed the differences of interaction strength of ligand-receptor pairs between CML and healthy controls and extracted the top 20 ligand-receptor pairs with the largest differences (Fig. 5D, E). Several elevated ligand-receptor pairs in CML were related to cancer development, such as ADRB2_VEGFB, which was involved in tumor angiogenesis,^39^ and BTLA_TNFRSF14, which was reported in immune regulation.^40^ Several decreased ligand-receptor pairs in CML, such as ANXA1_FPR2, were indicated to be involved in the remission of inflammation.^41^ These results indicate that there is a complex communication network between T cell subsets and bone marrow microenvironment cells.

### Neutrophil subset is associated with CD8 TE expansion in CML

**Figure 6:**
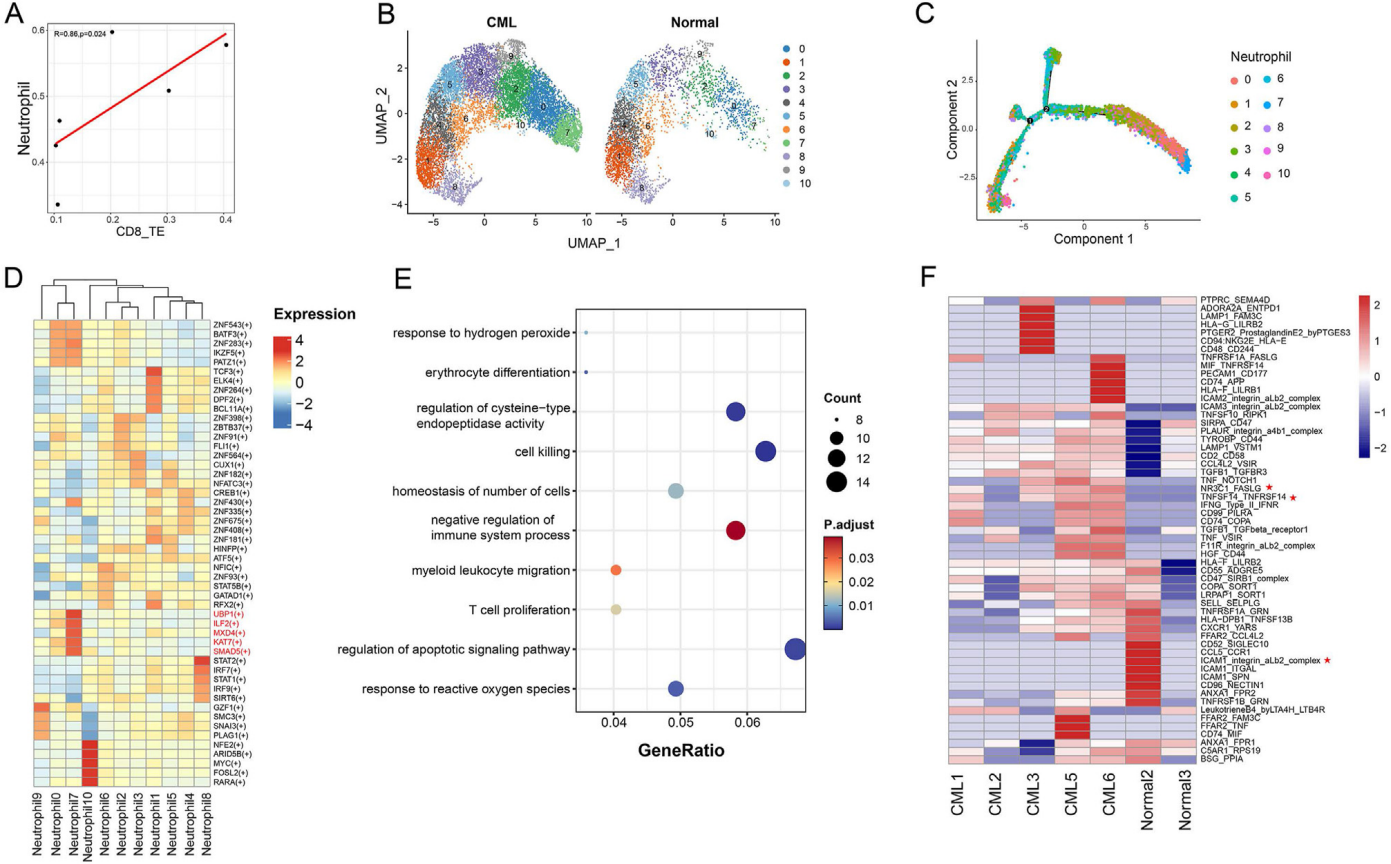
Neutrophil-7 and CD8 TE cells co-regulate the development of CML. **(A)** The scatter plots showing the correlation between CD8 TE cells and the proportion of neutrophils. **(B)** The UMAP plot showing the clustering of neutrophils in CML and healthy samples. **(C)** Pseudotime trajectory of neutrophils in all samples by Monocle2. **(D)** The heatmap showing the normalized expression of top five transcription factor regulons in each neutrophil cluster predicted by pySCENIC. **(E)** The bubble plot showing the biological function enrichment of differential genes between neutrophil-7 in all samples. **(F)** The heatmap showing the difference of cell–cell communication between CD8 TE cells and neutrophil-7 in CML and control samples. Stars indicate significantly different ligand-receptor pairs. CML, chronic myeloid leukemia; TE, terminal effector.

As neutrophils account for a relatively high proportion in the bone marrow and have been reported to be related to T cell functions,^42^^,^^43^ we examined the correlation between the proportions of neutrophils and CD8 TE cells in the bone marrow samples and found that CD8 TE cells was significantly correlated with neutrophils (Fig. 6A). We further subdivided neutrophils into 11 subtypes, among which neutrophil-7 was significantly enriched (*p* = 0.015) in CML patients (Fig. 6B; Fig. S4), suggesting that neutrophil-7 may be the subtype most associated with CD8 TE cells. To further explore the function of neutrophil-7, we used Monocle2 to perform pseudotime analysis. The cell differentiation trajectory exhibited that the neutrophil-7 was mostly located at the beginning of the differentiation trajectory (Fig. 6C). As cell differentiation is regulated by a complex transcription factor network,^44^ we used pySCENIC to evaluate the top five activated transcription factors in all neutrophil subtypes. We found that neutrophil-7 highly expressed the ILF2 transcription factor (Fig. 6D), which is essential for the expression of IL2 in T cells^45^ and is expressed more frequently in leukemia cell lines.^46^ Furthermore, GO enrichment analysis exhibited that the differentially expressed genes between neutrophil-7 and other subtypes were significantly enriched in apoptosis, T cell proliferation, and negative regulation of the immune system (Fig. 6E and Table S7). Subsequently, we explored the ligand-receptor interaction between CD8 TE cells and neutrophil-7 and found that the ligand-receptor pair NR3C1_FASLG, which can stimulate the expansion of effector T cells,^47^ was significantly different between CML patients and healthy samples (Fig. 6F). These results suggest a significant interaction between neutrophil-7 and CD8 TE cells and may jointly promote the occurrence and development of CML.

## Discussion

Single-cell sequencing technology can well characterize the T cell landscape in the leukemia bone marrow microenvironment. However, most previous studies in CML have mainly focused on bone marrow stem cells^10^ or mononuclear cell sequencing,^9^ and research on the immune microenvironment of CML remains at the flow cytometry^48^ or single-molecule level.^49^ Compared with other types of leukemia,^29^^,^^50^ CML lacks a comprehensive analysis of T cells in the bone marrow microenvironment. Therefore, in this study, we systematically analyzed the immune changes of CML from two levels and conducted a preliminary analysis of the interaction between bone marrow cells and T cells. In addition, our study provides a list of markers for future specific labeling of cells with similar functions.

Considering the important role of T cells in the immune system, we first performed a single-cell analysis of CD3-positive cells in CML and control bone marrow. The results showed obvious differences in T cell numbers across samples, but TCR diversity was reduced in all CML samples. The number of CD4 T cells in CML showed a downward trend, while CD8 T cells increased significantly. Furthermore, CD8 T cells have increased cytotoxicity scores and express higher levels of immunosuppressive signatures. This indicates that CML may induce enhanced T cell toxicity and an immunosuppressive state. Further analysis showed that the gene expression and cellular functions of T cells also changed significantly in CML.

By further subdividing T cell subsets, we found that CD4 naïve cells were significantly decreased in CML samples, while CD8 TE cells were significantly elevated in CML samples. Despite the elevated levels of CD8 TE cells, these effector T cells may be gradually exhausted due to continued exposure to the CML microenvironment.^30^ This could be the reason why leukemia patients have weaker immune systems.^51^^,^^52^ Paired T cell subsets and TCR profiles can more accurately assess the functional properties of T cells.^29^ We observed that TCR clonotype and diversity in CML were lower than those in control samples, indicating that CML bone marrow is characterized by immunosuppression, which is consistent with previous studies.^53^ After mapping TCR profiles into T cell subsets, we found that TCR amplification was mainly concentrated in CD8 TE and CD4 TE cells. Given that the proportion of CD8 TE cells was significantly increased in CML patients, we speculate that CD8 TE cells may be an important factor causing the immune difference between CML patients and healthy people. Through differential gene expression analysis of single cells, we found that the differential genes of CD8 TE cells had many overlaps with CD8 T cells, indicating that CD8 TE cells may be the main reason for the differences in CD8 T cells. To identify the key genes, we mapped these differential genes to a bulk data set containing CML patients, healthy people, and patients who achieved remission after treatment, and found some genes were related to CML treatment, previously reported to play an important role in cancer progression; for example, IL7R can affect the development of T cells, and its mutations can induce the occurrence of disease.^54^

Previous reports have shown that the bone marrow microenvironment of CML will undergo significant changes.^55^, ^56^, ^57^ To explore the potential impact of the bone marrow microenvironment on T cells, we profiled the whole bone marrow cell atlas of the corresponding sample and merged it with a T cell map for cell–cell communication analysis. We found that in CML, the interaction frequency between neutrophils, mast cells, erythroblasts, CD14 monocytes, and CD8 TE cells was higher than that in control samples, while the interaction frequency between CD8 TE and pre B cells was lower than that in control samples. These cell–cell interactions have been revealed in immune regulation and diseases.^58^, ^59^, ^60^, ^61^, ^62^, ^63^, ^64^ Notably, multiple ligand-receptor pairs mediated these interactions, further indicating the potentially important impact of the bone marrow microenvironment on T cell dynamics.

The strength of cell interactions is affected by cell number.^65^ As neutrophils account for a relatively high proportion in the bone marrow, we observed a significant positive correlation between CD8 TE cells and neutrophils. Further subdivision analysis revealed that neutrophil-7 was specifically enriched in CML patients and exhibited the strongest cell–cell interactions with CD8 TE cells. Neutrophil-7 significantly overexpressed transcription factors such as ILF2, MXD4, and KAT7. Previous studies have found that in myeloid leukemia, targeting MXD4 can eliminate leukemia-initiating cells,^66^ and KAT7 expression is elevated in leukemia cells. Knocking out KAT7 reduces the viability and proliferation of leukemia cells and induces cell apoptosis.^67^^,^^68^ Analysis of the ligand-receptor interaction between neutrophil-7 and CD8 TE cells revealed that there were significant differences of ligand-receptor pairs such as NR3C1_FASLG and TNFSF14_TNFRSF14 between CML patients and healthy samples, among which NR3C1_FASLG stimulated the expansion of effector T cells and TNFRSF14 promoted cytotoxicity,^69^ which is consistent with our results. These results suggest a potential regulatory relationship between neutrophil-7 and CD8 TE cells in the CML bone marrow microenvironment.

In conclusion, we performed multi-level single-cell sequencing to comprehensively characterize the T cell subsets of CML patients and healthy control samples, revealing significant changes in the number and gene expression of T cells, diversity of TCR repertoire, and cell–cell communication network. Our results demonstrate the expansion and diversity of CD8TE cells in CML and the potential relationship between bone marrow microenvironment cells and T cell subsets, providing a valuable resource to understand the immune changes in CML.

## CRediT authorship contribution statement

**Chenjian Zhuo:** Writing – original draft, Formal analysis. **Xin Dong:** Formal analysis. **Xueya Zhao:** Data curation. **Weiru Wu:** Resources. **Hao Zhou:** Resources. **Jing Feng:** Methodology. **Lingbo Liu:** Supervision. **Mingqian Feng:** Supervision. **Chunjiang He:** Writing – review & editing, Supervision, Conceptualization. **Yu Hou:** Writing – review & editing, Supervision, Conceptualization.

## Ethics declaration

The study was approved by the Ethics Committee (Institutional Review Board) of Third Military Medical University (Army Medical University) Southwest Hospital (No. KY2020199). All patients provided written informed consent for participation according to the Declaration of Helsinki and written informed consent for publication. The clinical information was retrieved from the medical records.

## Data availability

The raw data of single-cell sequencing can be accessed through the GEO database (GSE241842).

## Funding

This research was funded by the 10.13039/501100001809National Natural Science Foundation of China (No. 82170115 and 82170170) and the Chongqing Science Fund for Distinguished Young Scholars (CSTB2022NSCQ-JQX0032).

## Conflict of interests

The authors declared no conflict of interests.
