# Supplementary material for: Single-cell sequencing reveals the expansion and diversity of T cell subsets in the bone marrow microenvironment of chronic myeloid leukemia
Source: Genes Dis. 2025 Apr 4;12(5):101626. doi: 10.1016/j.gendis.2025.101626 (PMC12221761; doi:10.1016/j.gendis.2025.101626)
Supplement: Multimedia component 1 [file mmc1.docx]

**Supplemental Legends**

**Figure S1. Batch effect removing and clustering of T cell.** A. UMAP plot shows the effect of removing batches for 8 samples. B. UMAP plot shows clustering results of T cells. C. UMAP map shows the distribution of CD4 T cells and CD8 T cells.

**Figure S2. Composition and functional variation of CD4 T cells in CML.**A. Boxplot shows the cytotoxicity scores of CD4 T cells between CML and healthy controls. B. Heatmap shows differentially expressed genes of CD4 T cells between CML and healthy controls. C. Bubble plot shows the biological functions of differentially expressed genes in CD4 T cells.

**Figure S3. The annotation of bone marrow microenvironment cells.** A. Dot plot showing the expression of marker genes in 16 bone marrow cells. B. UMAP diagram showing cell dimensionality reduction and annotation.

**Figure S4. The proportion of neutrophil subtypes in bone marrow of CML and healthy samples. Ns: no significance.**

**Table S1. Statistic information of CML and healthy controls.**

**Table S2. Gene markers for cell annotation.**

**Table S3. Differential expression genes of CD8 T cells between CML and healthy controls.**

**Table S4. Differential expression genes of CD4 T cells between CML and healthy controls.**

**Table S5. Differential expression genes of CD8 TE cells between CML and healthy controls.**

**Table S6. Differential expression genes specifically present in CD8 TE cells between CML and healthy controls.**

**Table S7. Differential expressed genes between neutrophil clusters.**
