# Supplementary figures and images for: Single-cell sequencing reveals the expansion and diversity of T cell subsets in the bone marrow microenvironment of chronic myeloid leukemia
Source: Genes Dis. 2025 Apr 4;12(5):101626. doi: 10.1016/j.gendis.2025.101626 (PMC12221761; doi:10.1016/j.gendis.2025.101626)

**A**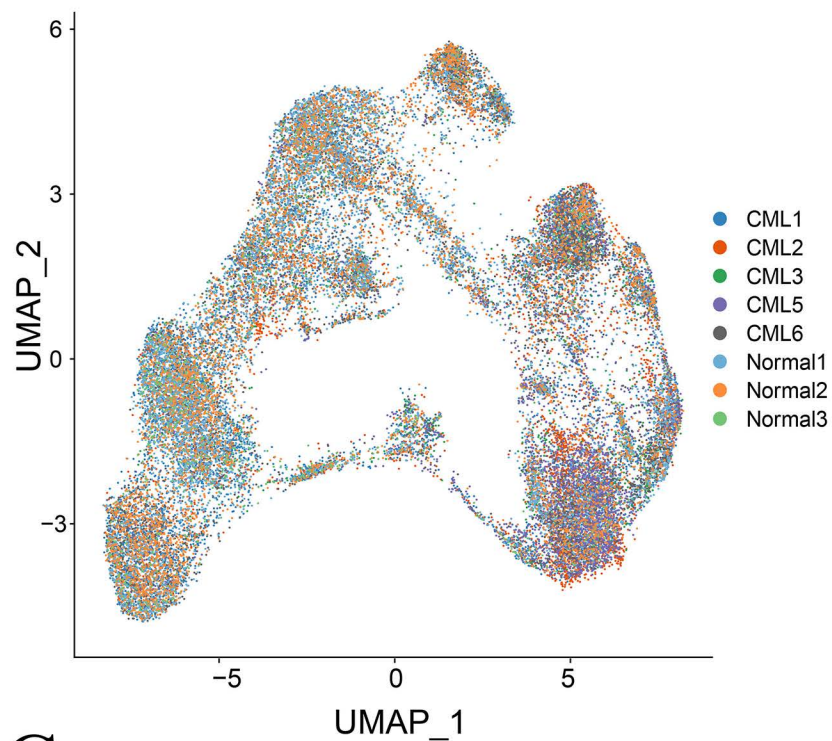**B**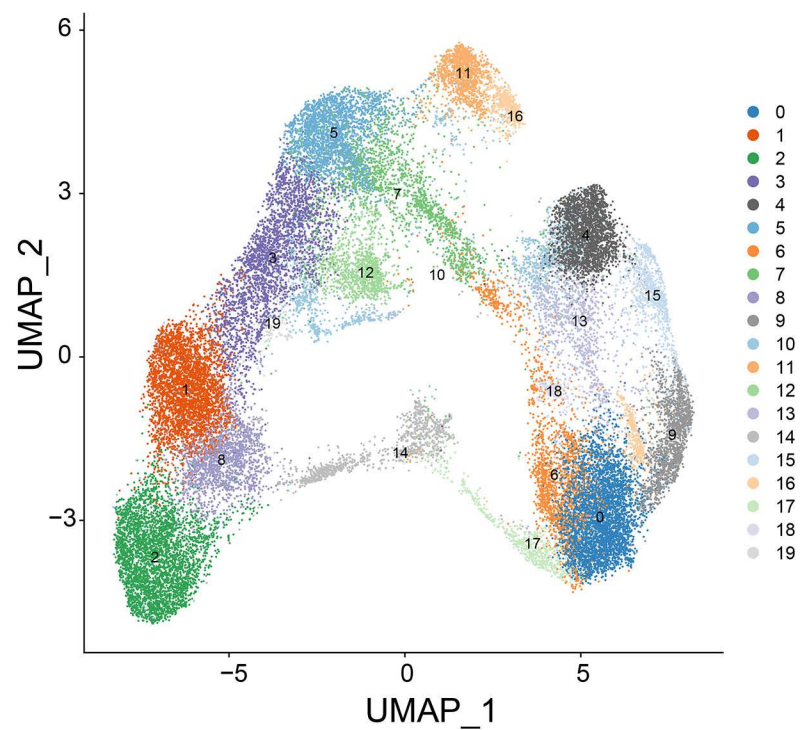**C**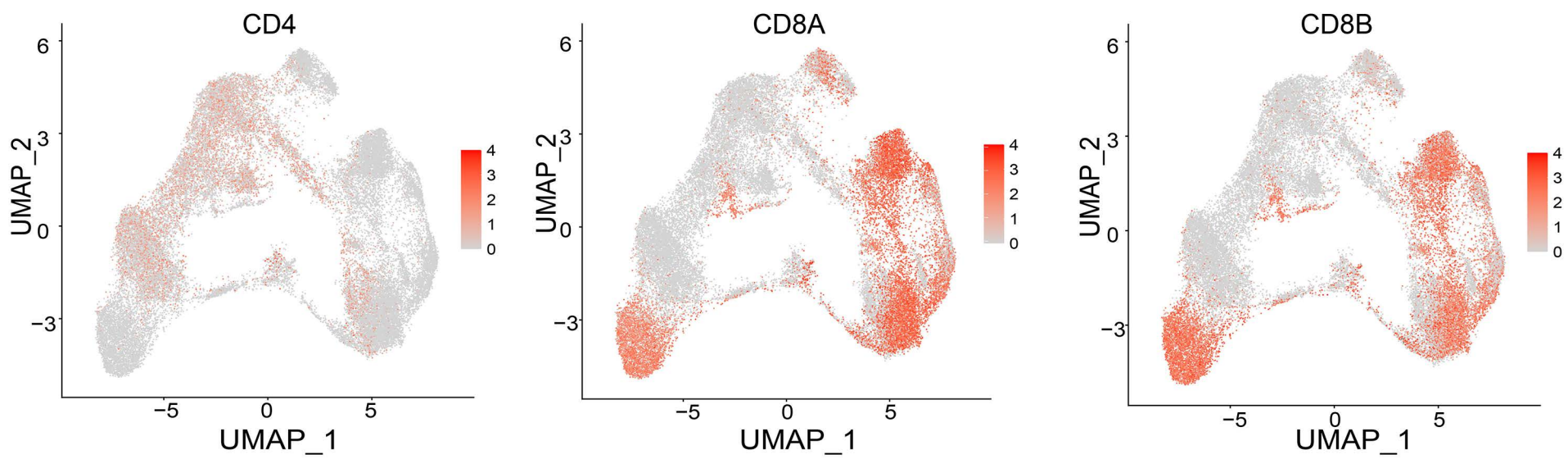

Supplement: Multimedia component 2 [file mmc2.pdf]

A

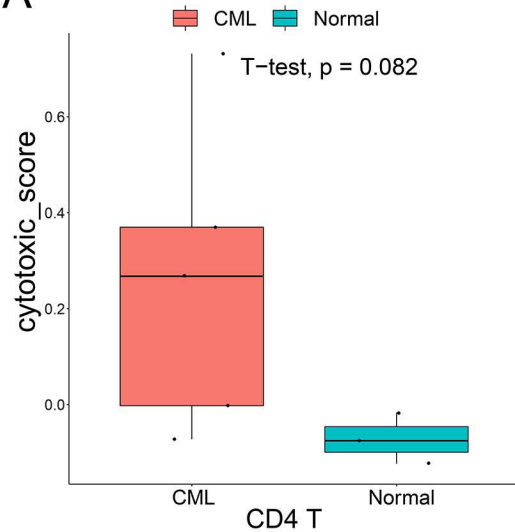

B

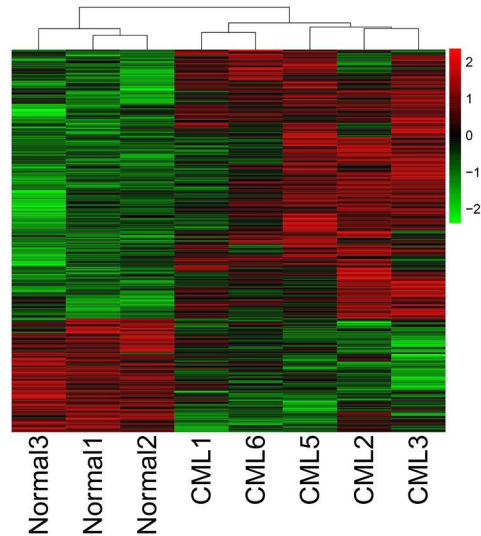

C

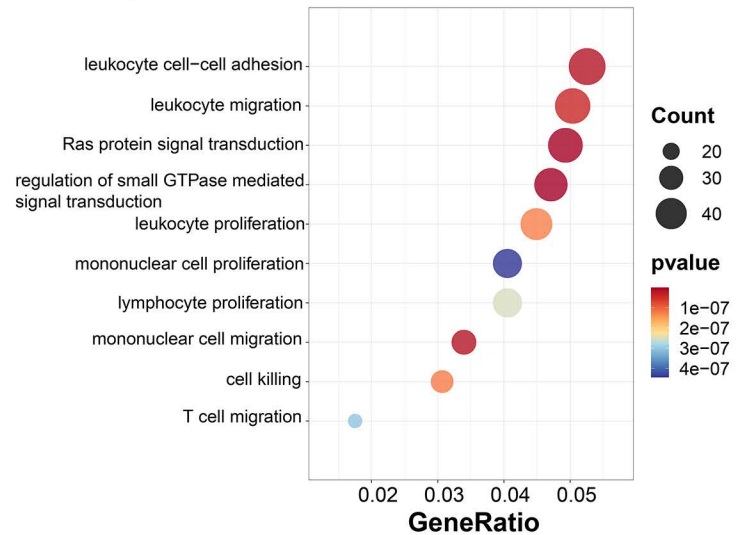

Supplement: Multimedia component 3 [file mmc3.pdf]

A

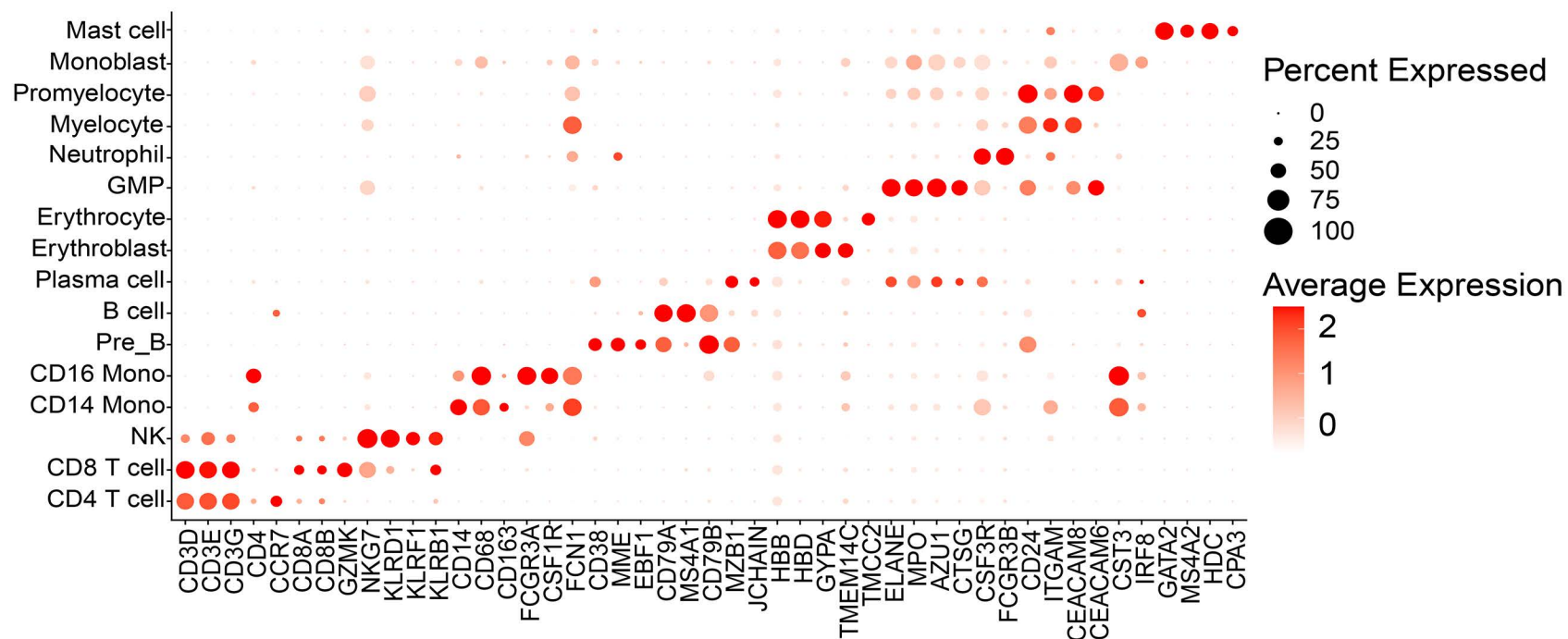

B

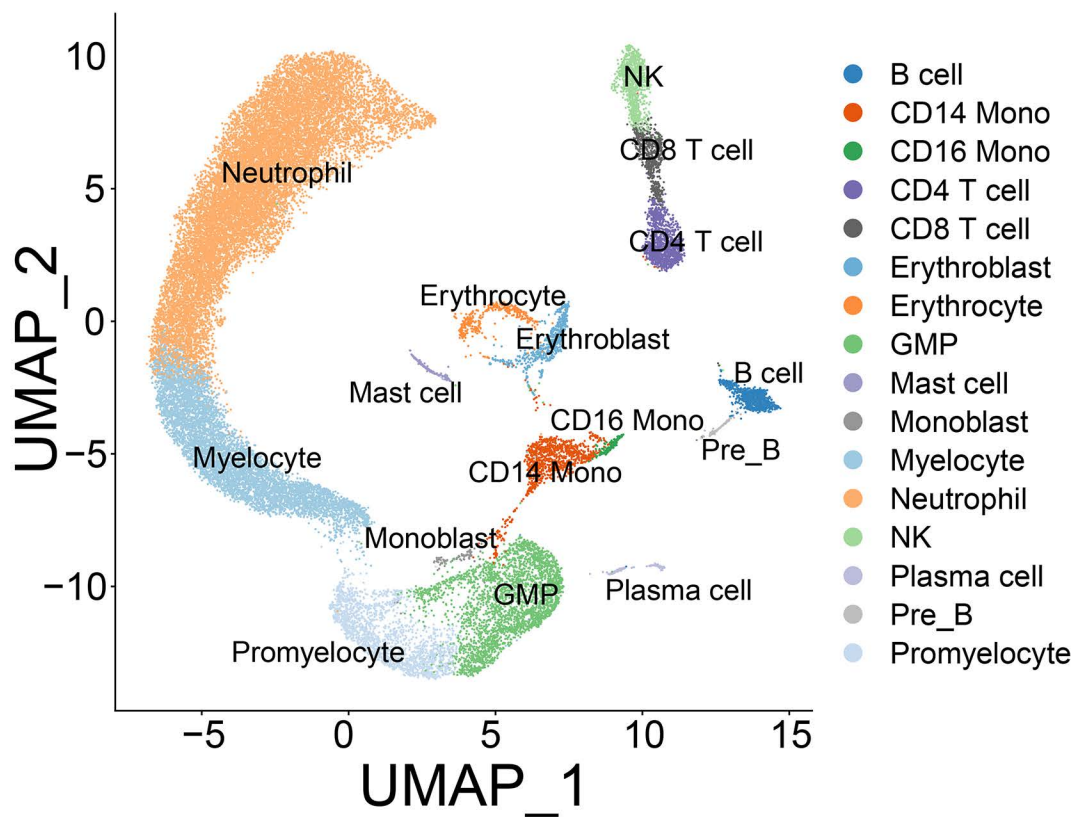

Supplement: Multimedia component 4 [file mmc4.pdf]

Percentage

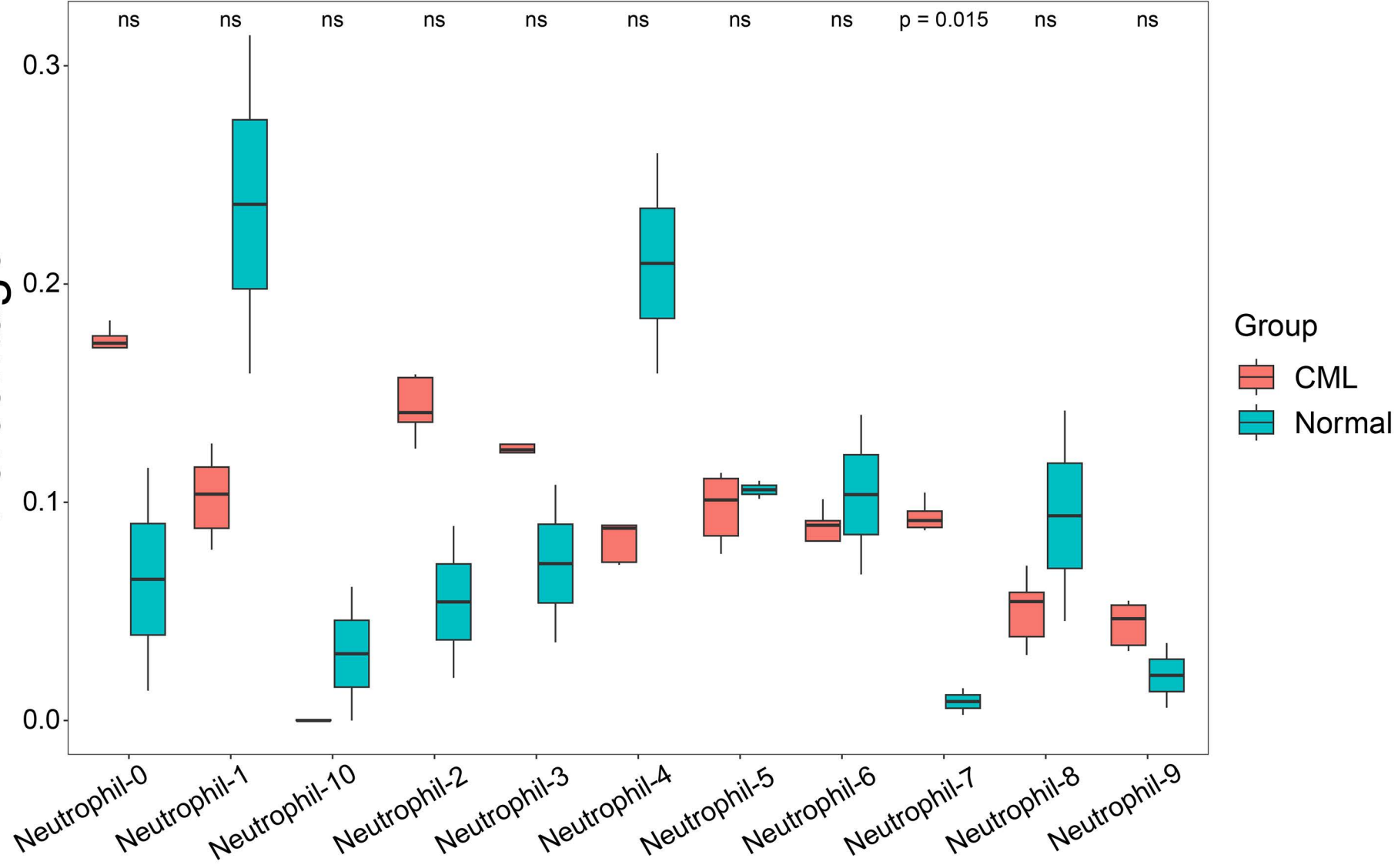

Supplement: Multimedia component 5 [file mmc5.pdf]
